# The Salaries of Hospital Officers

**Published:** 1920-02-14

**Authors:** 


### THE EDITOR'S LETTER-BOX.

### THE SALARIES OF HOSPITAL OFFICERS.

*To the Editor of* The Hospital.

Sir,—I am sure all hospital workers will have noted your remarks that you have recently published as to salaries being offered at the present day by committees of our voluntary hospitals with satisfaction. Recently we have had a provincial hospital offering £350 per annum for a secretary; another provincial institution advertising for an official in the steward's department at £135 per annum. Now to-day a secretary is required for one of our large general hospitals at £500 per annum, equal to about £250 per annum pre-war. No wonder this sort of thing fails to attract suitable men to a hospital career, when emoluments are offered that a skilled manual worker would scorn with contempt. Brains are evidently very cheap in these days of high cost of living, and high everything else.

Hospital officers have little chance of saving for their families and old age, and in most cases no security for a pension; although much ink has been spilt and many conferences held, no definite scheme of pensions has yet been evolved. The authorities of King Edward's Hospital Fund have always taken the greatest interest in the London hospitals, so one might venture to ask, will they take up the question of salaries paid to all engaged in a secretarial and clerical capacity, with a view, if possible, of something being done to improve their status from a financial point of view?

I enclose my card, and beg to sign myself,

Yours obediently,

Hospital Officer.

